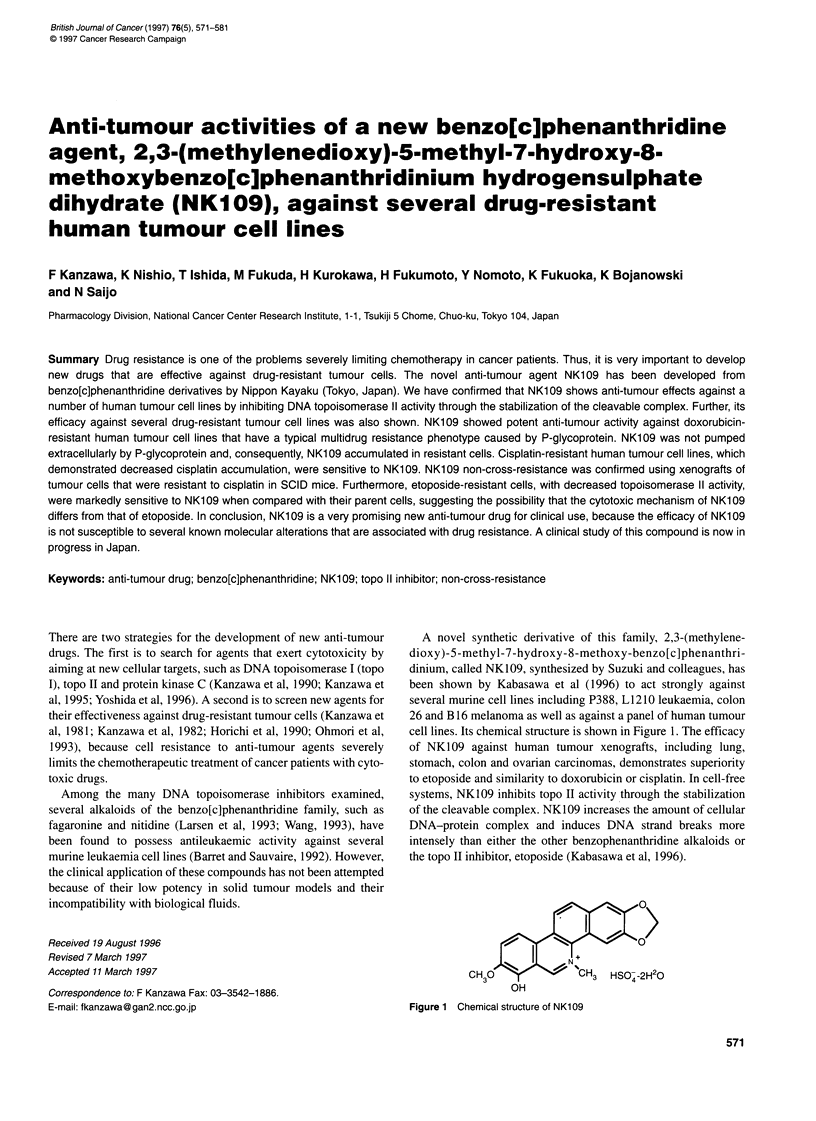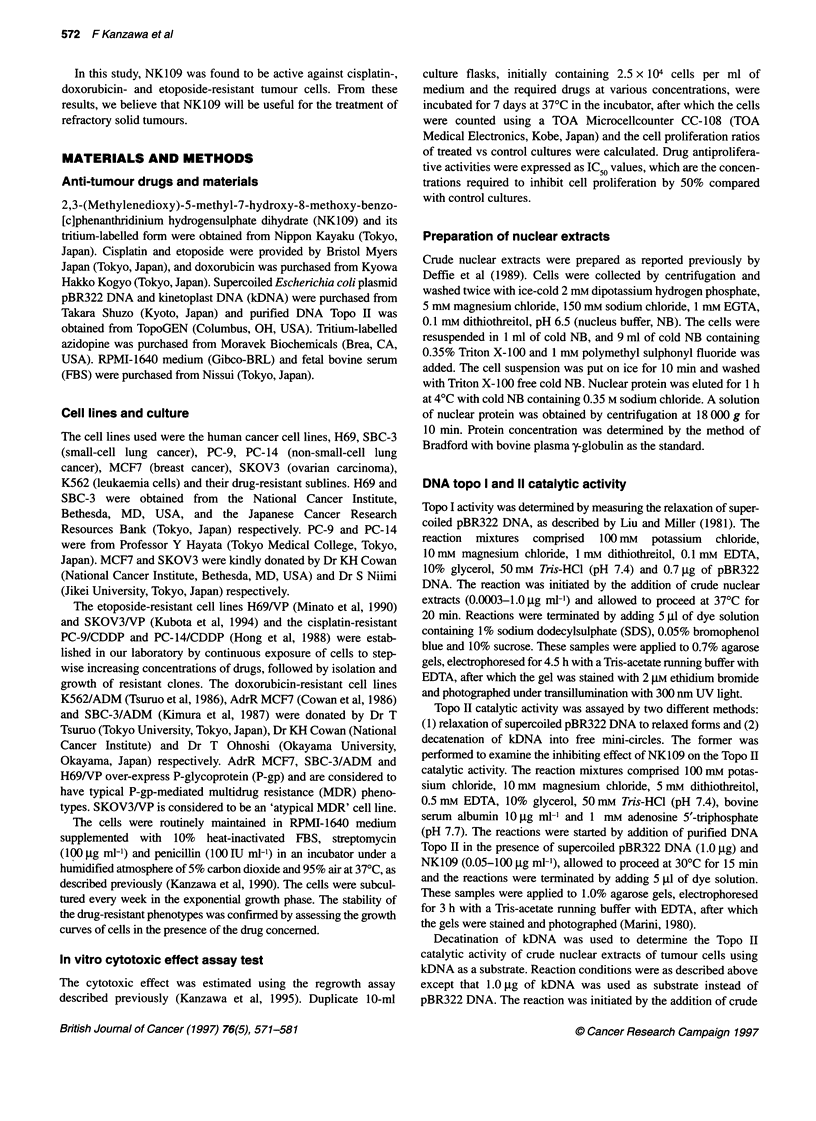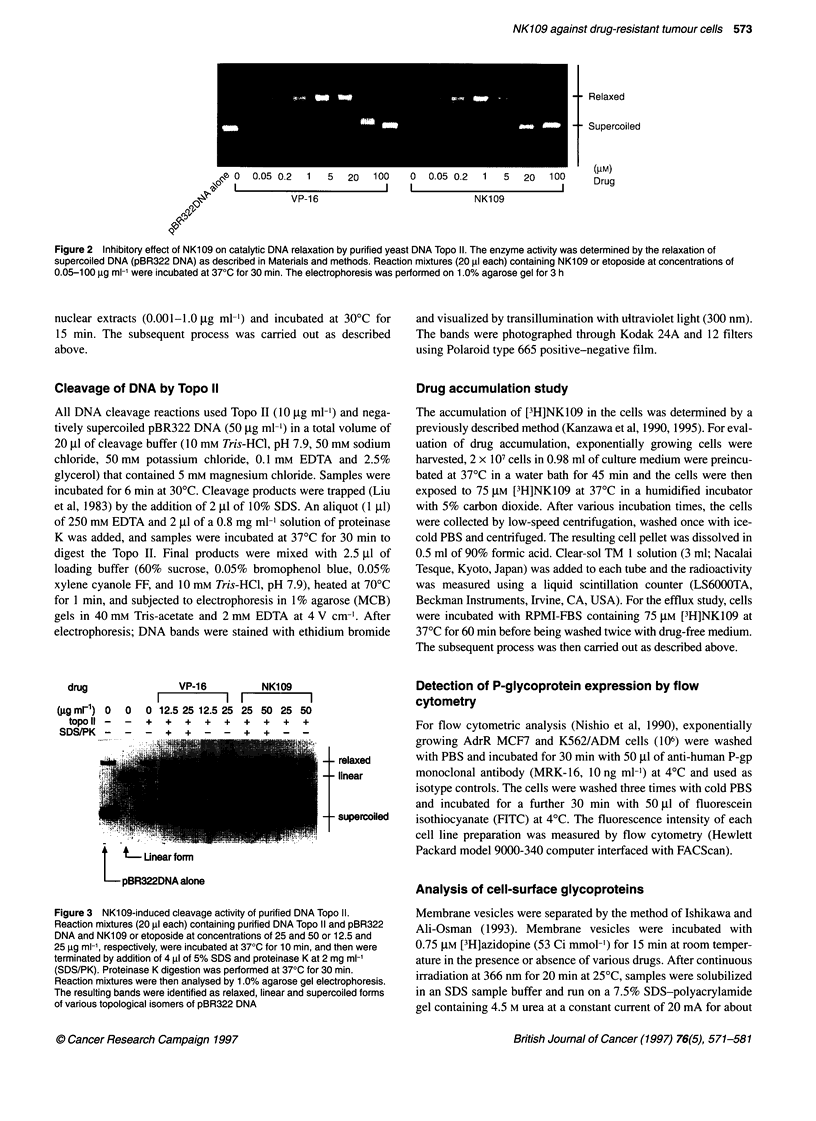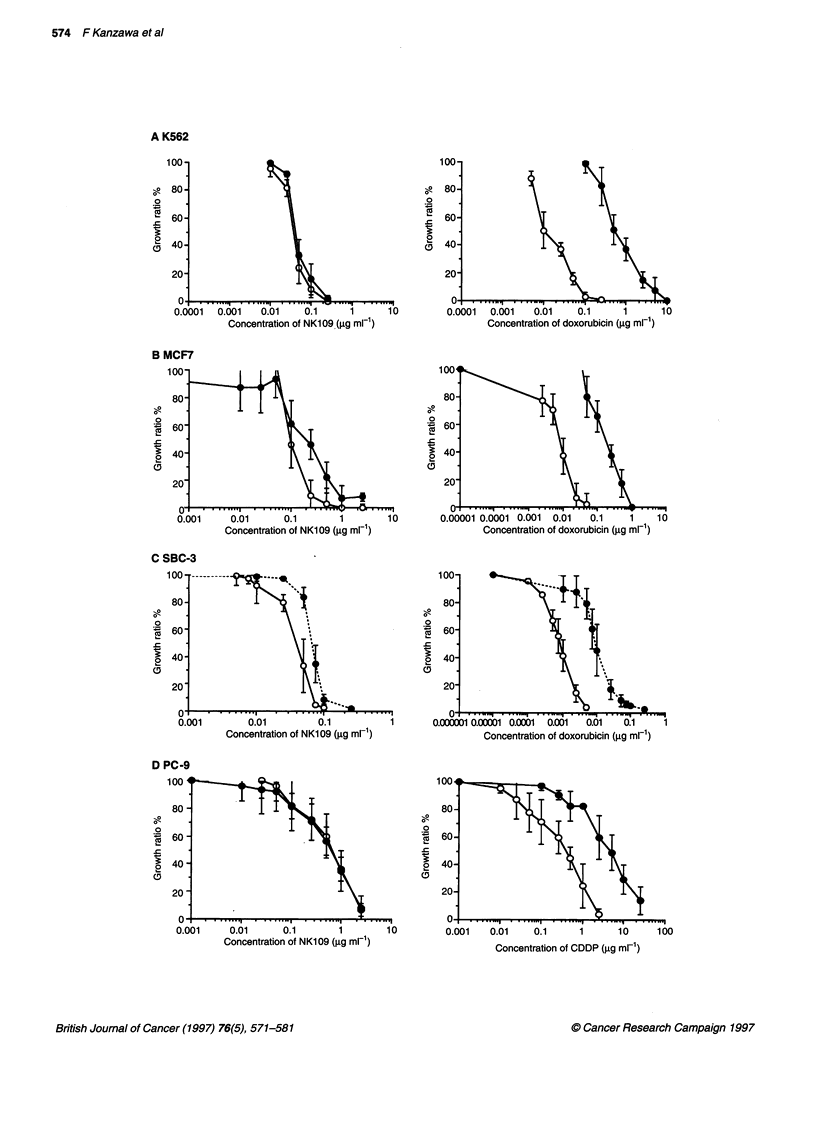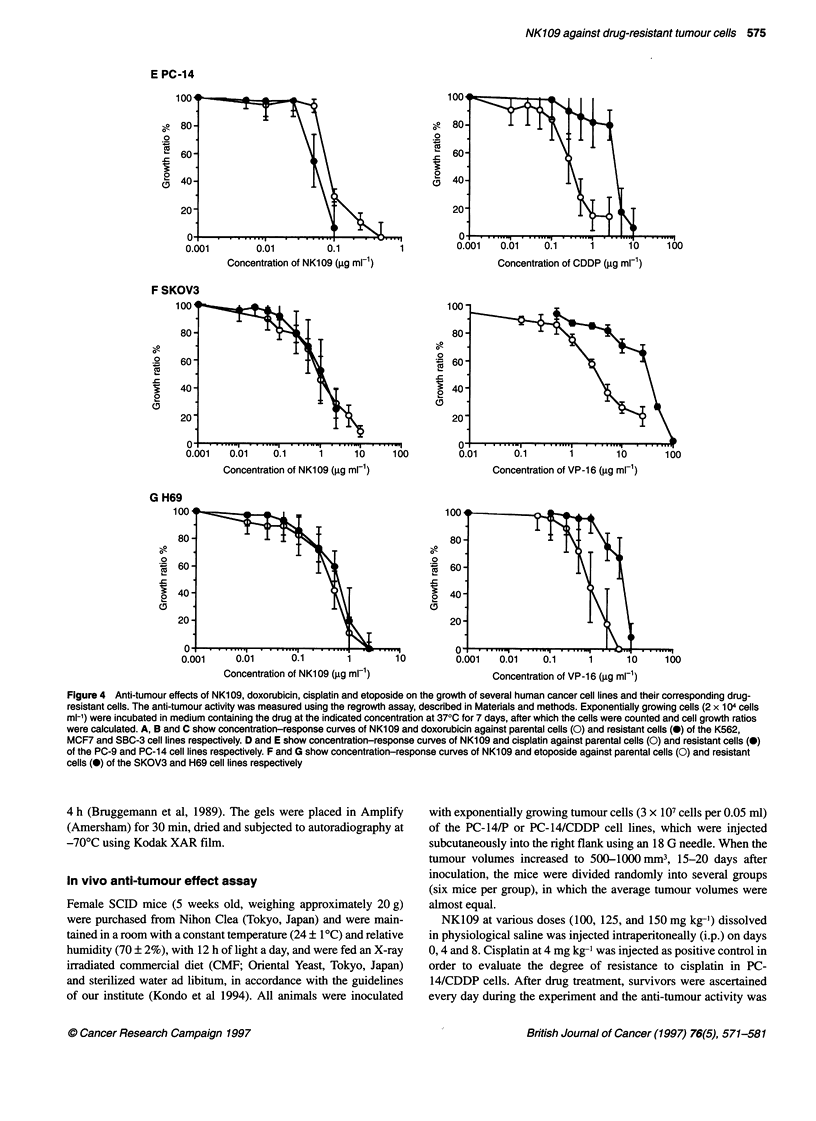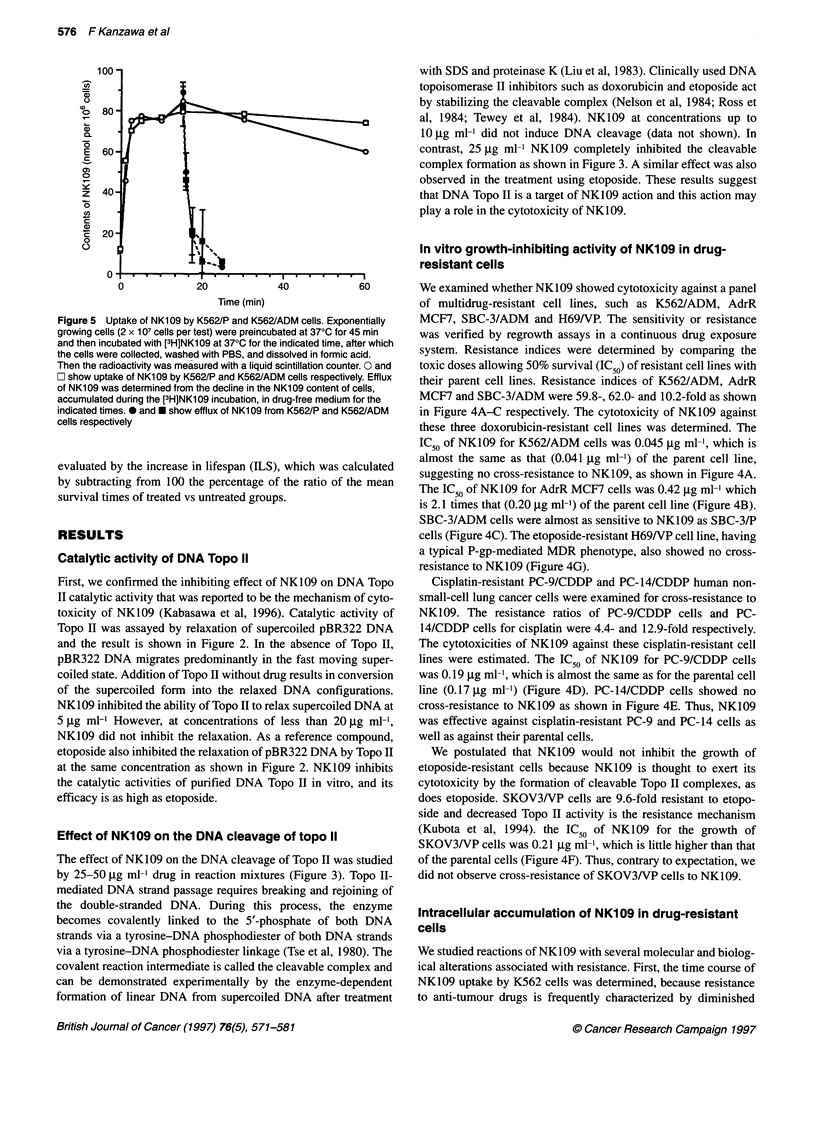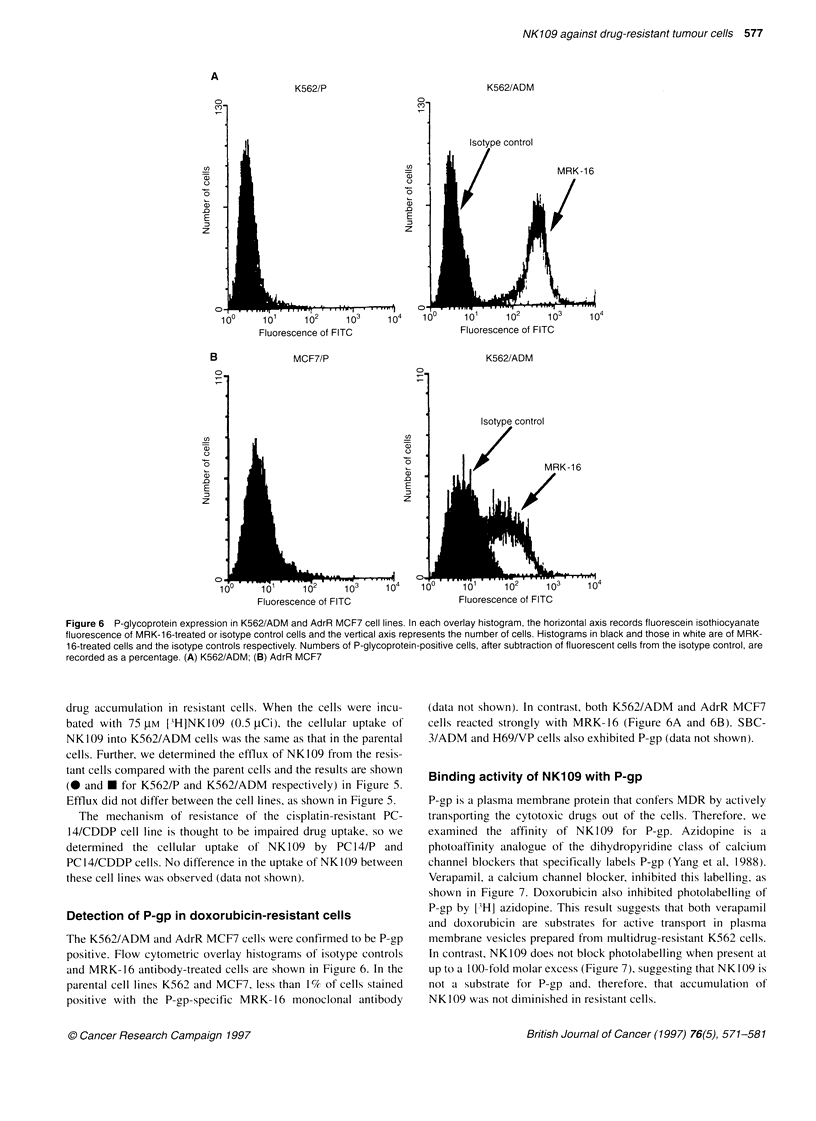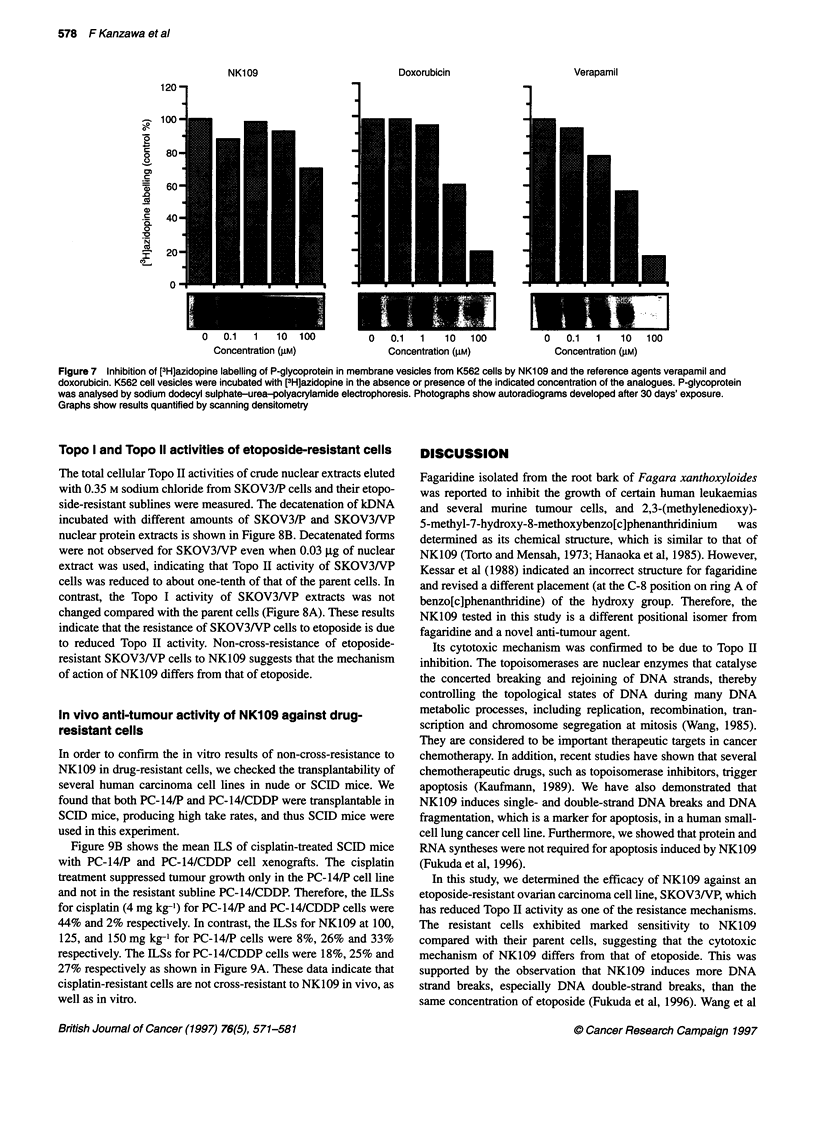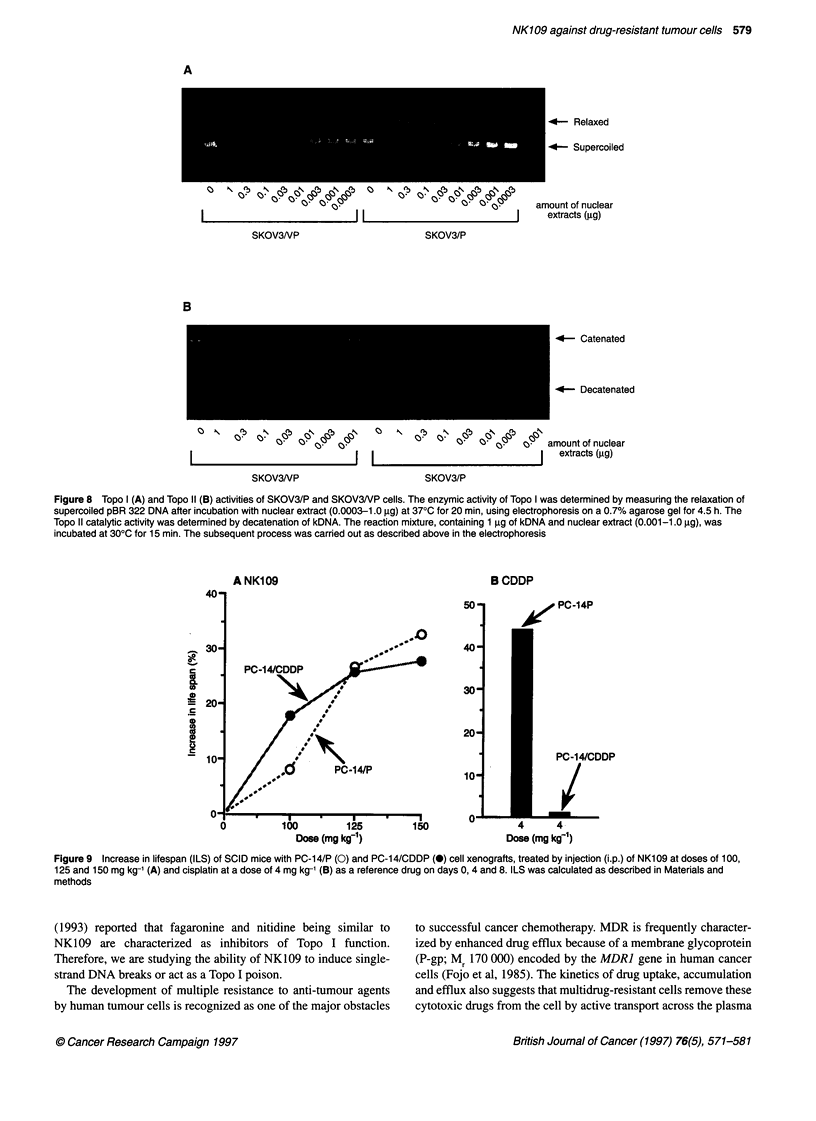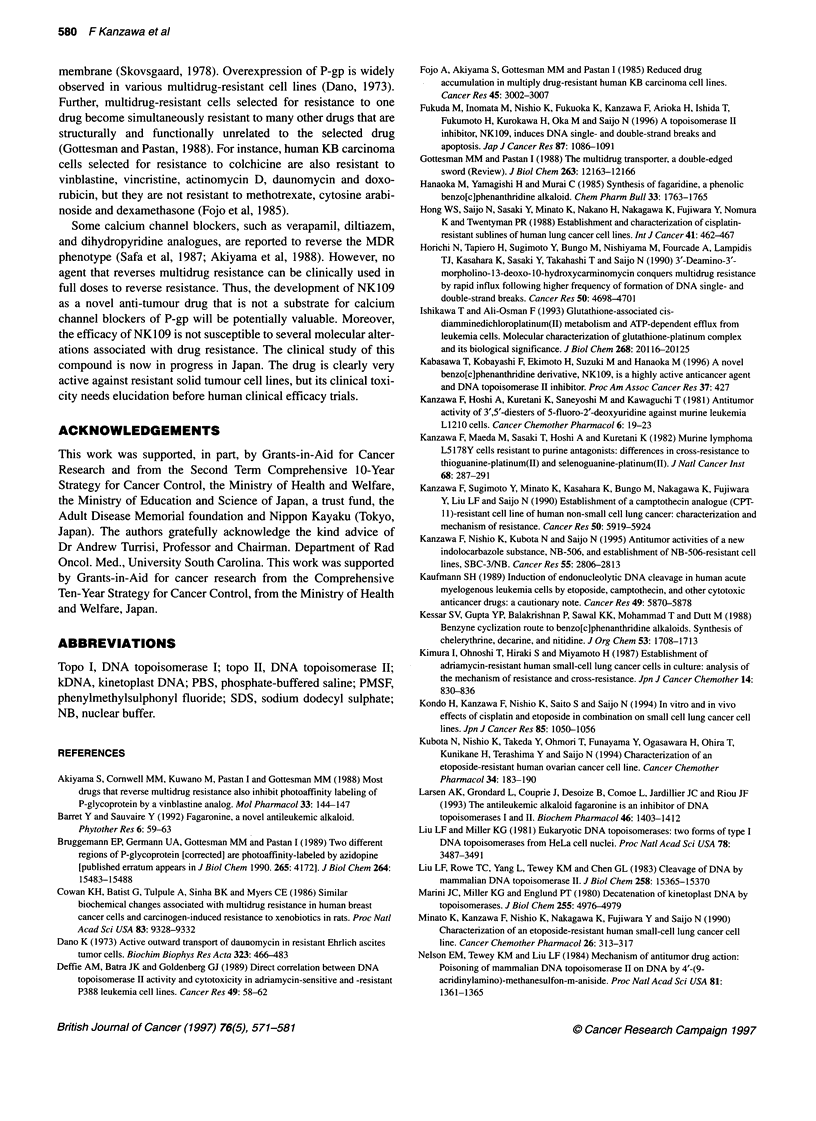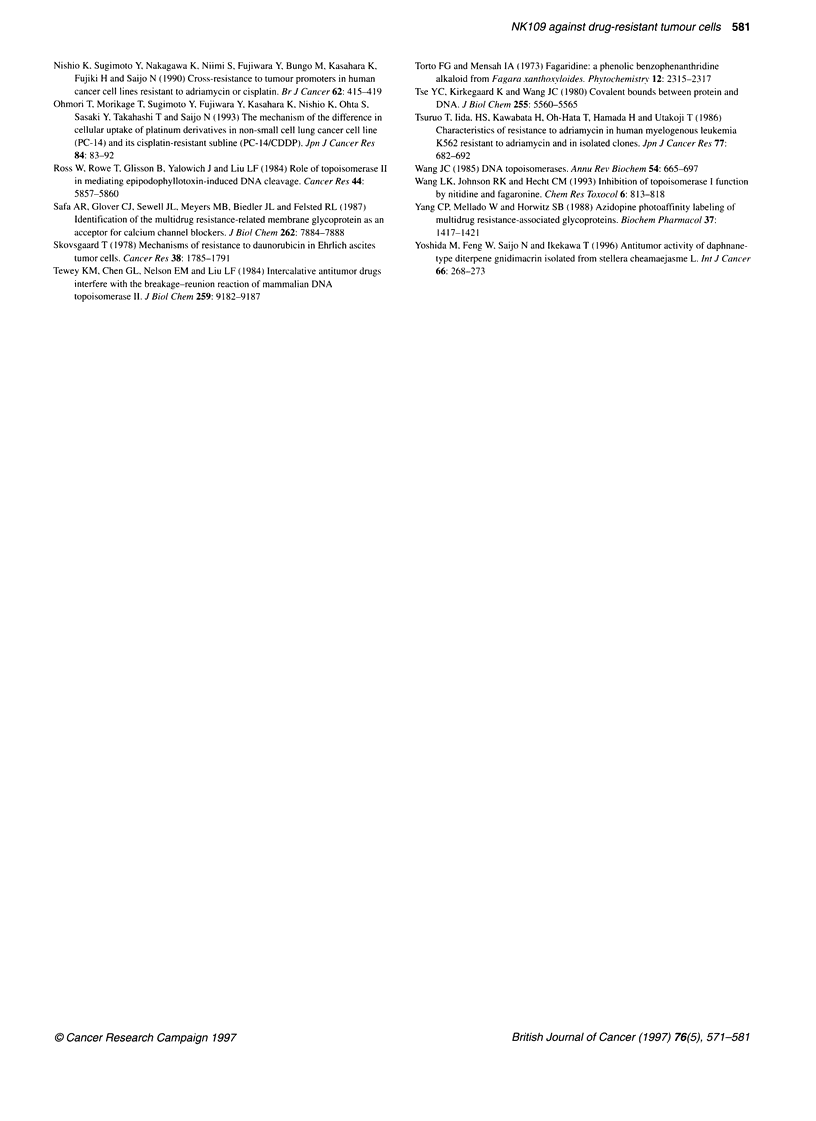# Anti-tumour activities of a new benzo[c]phenanthridine agent, 2,3-(methylenedioxy)-5-methyl-7-hydroxy-8-methoxybenzo[c]phena nthridini um hydrogensulphate dihydrate (NK109), against several drug-resistant human tumour cell lines.

**DOI:** 10.1038/bjc.1997.428

**Published:** 1997

**Authors:** F. Kanzawa, K. Nishio, T. Ishida, M. Fukuda, H. Kurokawa, H. Fukumoto, Y. Nomoto, K. Fukuoka, K. Bojanowski, N. Saijo

**Affiliations:** Pharmacology Division, National Cancer Center Research Institute, Tokyo, Japan.

## Abstract

**Images:**


*British Journal of Cancer* (1997) **76**(5), 571–581
© 1997 Cancer Research Campaign

### Anti-tumour activities of a new benzo[c]phenanthridine agent, 2,3-(methylenedioxy)-5-methyl-7-hydroxy-8-methoxybenzo[c]phenanthridinium hydrogensulphate dihydrate (NK109), against several drug-resistant human tumour cell lines

**F Kanzawa, K Nishio, T Ishida, M Fukuda, H Kurokawa, H Fukumoto, Y Nomoto, K Fukuoka, K Bojanowski and N Saijo**

Pharmacology Division, National Cancer Center Research Institute, 1-1, Tsukiji 5 Chome, Chuo-ku, Tokyo 104, Japan

**Summary** Drug resistance is one of the problems severely limiting chemotherapy in cancer patients. Thus, it is very important to develop new drugs that are effective against drug-resistant tumour cells. The novel anti-tumour agent NK109 has been developed from benzo[c]phenanthridine derivatives by Nippon Kayaku (Tokyo, Japan). We have confirmed that NK109 shows anti-tumour effects against a number of human tumour cell lines by inhibiting DNA topoisomerase II activity through the stabilization of the cleavable complex. Further, its efficacy against several drug-resistant tumour cell lines was also shown. NK109 showed potent anti-tumour activity against doxorubicin-resistant human tumour cell lines that have a typical multidrug resistance phenotype caused by P-glycoprotein. NK109 was not pumped extracellularly by P-glycoprotein and, consequently, NK109 accumulated in resistant cells. Cisplatin-resistant human tumour cell lines, which demonstrated decreased cisplatin accumulation, were sensitive to NK109. NK109 non-cross-resistance was confirmed using xenografts of tumour cells that were resistant to cisplatin in SCID mice. Furthermore, etoposide-resistant cells, with decreased topoisomerase II activity, were markedly sensitive to NK109 when compared with their parent cells, suggesting the possibility that the cytotoxic mechanism of NK109 differs from that of etoposide. In conclusion, NK109 is a very promising new anti-tumour drug for clinical use, because the efficacy of NK109 is not susceptible to several known molecular alterations that are associated with drug resistance. A clinical study of this compound is now in progress in Japan.

**Keywords:** anti-tumour drug; benzo[c]phenanthridine; NK109; topo II inhibitor; non-cross-resistance

There are two strategies for the development of new anti-tumour drugs. The first is to search for agents that exert cytotoxicity by aiming at new cellular targets, such as DNA topoisomerase I (topo I), topo II and protein kinase C (Kanzawa et al, 1990; Kanzawa et al, 1995; Yoshida et al, 1996). A second is to screen new agents for their effectiveness against drug-resistant tumour cells (Kanzawa et al, 1981; Kanzawa et al, 1982; Horichi et al, 1990; Ohmori et al, 1993), because cell resistance to anti-tumour agents severely limits the chemotherapeutic treatment of cancer patients with cytotoxic drugs.

Among the many DNA topoisomerase inhibitors examined, several alkaloids of the benzo[c]phenanthridine family, such as fagaronine and nitidine (Larsen et al, 1993; Wang, 1993), have been found to possess antileukaemic activity against several murine leukaemia cell lines (Barret and Sauvaire, 1992). However, the clinical application of these compounds has not been attempted because of their low potency in solid tumour models and their incompatibility with biological fluids.

*Received 19 August 1996*
*Revised 7 March 1997*
*Accepted 11 March 1997*

*Correspondence to:* F Kanzawa Fax: 03–3542–1886.
E-mail: fkanzawa@gan2.ncc.go.jp

A novel synthetic derivative of this family, 2,3-(methylenedioxy)-5-methyl-7-hydroxy-8-methoxy-benzo[c]phenanthridinium, called NK109, synthesized by Suzuki and colleagues, has been shown by Kabasawa et al (1996) to act strongly against several murine cell lines including P388, L1210 leukaemia, colon 26 and B16 melanoma as well as against a panel of human tumour cell lines. Its chemical structure is shown in Figure 1. The efficacy of NK109 against human tumour xenografts, including lung, stomach, colon and ovarian carcinomas, demonstrates superiority to etoposide and similarity to doxorubicin or cisplatin. In cell-free systems, NK109 inhibits topo II activity through the stabilization of the cleavable complex. NK109 increases the amount of cellular DNA–protein complex and induces DNA strand breaks more intensely than either the other benzophenanthridine alkaloids or the topo II inhibitor, etoposide (Kabasawa et al, 1996).

**Figure 1** Chemical structure of NK109

In this study, NK109 was found to be active against cisplatin-, doxorubicin- and etoposide-resistant tumour cells. From these results, we believe that NK109 will be useful for the treatment of refractory solid tumours.

### MATERIALS AND METHODS

### Anti-tumour drugs and materials

2,3-(Methylenedioxy)-5-methyl-7-hydroxy-8-methoxy-benzo-[c]phenanthridinium hydrogensulphate dihydrate (NK109) and its tritium-labelled form were obtained from Nippon Kayaku (Tokyo, Japan). Cisplatin and etoposide were provided by Bristol Myers Japan (Tokyo, Japan), and doxorubicin was purchased from Kyowa Hakko Kogyo (Tokyo, Japan). Supercoiled *Escherichia coli* plasmid pBR322 DNA and kinetoplast DNA (kDNA) were purchased from Takara Shuzo (Kyoto, Japan) and purified DNA Topo II was obtained from TopoGEN (Columbus, OH, USA). Tritium-labelled azidopine was purchased from Moravek Biochemicals (Brea, CA, USA). RPMI-1640 medium (Gibco-BRL) and fetal bovine serum (FBS) were purchased from Nissui (Tokyo, Japan).

### Cell lines and culture

The cell lines used were the human cancer cell lines, H69, SBC-3 (small-cell lung cancer), PC-9, PC-14 (non-small-cell lung cancer), MCF7 (breast cancer), SKOV3 (ovarian carcinoma), K562 (leukaemia cells) and their drug-resistant sublines. H69 and SBC-3 were obtained from the National Cancer Institute, Bethesda, MD, USA, and the Japanese Cancer Research Resources Bank (Tokyo, Japan) respectively. PC-9 and PC-14 were from Professor Y Hayata (Tokyo Medical College, Tokyo, Japan). MCF7 and SKOV3 were kindly donated by Dr KH Cowan (National Cancer Institute, Bethesda, MD, USA) and Dr S Niimi (Jikei University, Tokyo, Japan) respectively.

The etoposide-resistant cell lines H69/VP (Minato et al, 1990) and SKOV3/VP (Kubota et al, 1994) and the cisplatin-resistant PC-9/CDDP and PC-14/CDDP (Hong et al, 1988) were established in our laboratory by continuous exposure of cells to stepwise increasing concentrations of drugs, followed by isolation and growth of resistant clones. The doxorubicin-resistant cell lines K562/ADM (Tsuruo et al, 1986), AdrR MCF7 (Cowan et al, 1986) and SBC-3/ADM (Kimura et al, 1987) were donated by Dr T Tsuruo (Tokyo University, Tokyo, Japan), Dr KH Cowan (National Cancer Institute) and Dr T Ohnoshi (Okayama University, Okayama, Japan) respectively. AdrR MCF7, SBC-3/ADM and H69/VP over-express P-glycoprotein (P-gp) and are considered to have typical P-gp-mediated multidrug resistance (MDR) phenotypes. SKOV3/VP is considered to be an 'atypical MDR' cell line.

The cells were routinely maintained in RPMI-1640 medium supplemented with 10% heat-inactivated FBS, streptomycin (100 μg $ml^{-1}$) and penicillin (100 IU $ml^{-1}$) in an incubator under a humidified atmosphere of 5% carbon dioxide and 95% air at 37°C, as described previously (Kanzawa et al, 1990). The cells were subcultured every week in the exponential growth phase. The stability of the drug-resistant phenotypes was confirmed by assessing the growth curves of cells in the presence of the drug concerned.

### In vitro cytotoxic effect assay test

The cytotoxic effect was estimated using the regrowth assay described previously (Kanzawa et al, 1995). Duplicate 10-ml culture flasks, initially containing $2.5 \times 10^4$ cells per ml of medium and the required drugs at various concentrations, were incubated for 7 days at 37°C in the incubator, after which the cells were counted using a TOA Microcellcounter CC-108 (TOA Medical Electronics, Kobe, Japan) and the cell proliferation ratios of treated vs control cultures were calculated. Drug antiproliferative activities were expressed as $IC_{50}$ values, which are the concentrations required to inhibit cell proliferation by 50% compared with control cultures.

### Preparation of nuclear extracts

Crude nuclear extracts were prepared as reported previously by Deffie et al (1989). Cells were collected by centrifugation and washed twice with ice-cold 2 mM dipotassium hydrogen phosphate, 5 mM magnesium chloride, 150 mM sodium chloride, 1 mM EGTA, 0.1 mM dithiothreitol, pH 6.5 (nucleus buffer, NB). The cells were resuspended in 1 ml of cold NB, and 9 ml of cold NB containing 0.35% Triton X-100 and 1 mM polymethyl sulphonyl fluoride was added. The cell suspension was put on ice for 10 min and washed with Triton X-100 free cold NB. Nuclear protein was eluted for 1 h at 4°C with cold NB containing 0.35 M sodium chloride. A solution of nuclear protein was obtained by centrifugation at 18 000 ***g*** for 10 min. Protein concentration was determined by the method of Bradford with bovine plasma γ-globulin as the standard.

### DNA topo I and II catalytic activity

Topo I activity was determined by measuring the relaxation of supercoiled pBR322 DNA, as described by Liu and Miller (1981). The reaction mixtures comprised 100 mM potassium chloride, 10 mM magnesium chloride, 1 mM dithiothreitol, 0.1 mM EDTA, 10% glycerol, 50 mM *Tris*-HCl (pH 7.4) and 0.7 μg of pBR322 DNA. The reaction was initiated by the addition of crude nuclear extracts (0.0003–1.0 μg $ml^{-1}$) and allowed to proceed at 37°C for 20 min. Reactions were terminated by adding 5 μl of dye solution containing 1% sodium dodecylsulphate (SDS), 0.05% bromophenol blue and 10% sucrose. These samples were applied to 0.7% agarose gels, electrophoresed for 4.5 h with a Tris-acetate running buffer with EDTA, after which the gel was stained with 2 μM ethidium bromide and photographed under transillumination with 300 nm UV light.

Topo II catalytic activity was assayed by two different methods: (1) relaxation of supercoiled pBR322 DNA to relaxed forms and (2) decatenation of kDNA into free mini-circles. The former was performed to examine the inhibiting effect of NK109 on the Topo II catalytic activity. The reaction mixtures comprised 100 mM potassium chloride, 10 mM magnesium chloride, 5 mM dithiothreitol, 0.5 mM EDTA, 10% glycerol, 50 mM *Tris*-HCl (pH 7.4), bovine serum albumin 10 μg $ml^{-1}$ and 1 mM adenosine 5′-triphosphate (pH 7.7). The reactions were started by addition of purified DNA Topo II in the presence of supercoiled pBR322 DNA (1.0 μg) and NK109 (0.05–100 μg $ml^{-1}$), allowed to proceed at 30°C for 15 min and the reactions were terminated by adding 5 μl of dye solution. These samples were applied to 1.0% agarose gels, electrophoresed for 3 h with a Tris-acetate running buffer with EDTA, after which the gels were stained and photographed (Marini, 1980).

Decatination of kDNA was used to determine the Topo II catalytic activity of crude nuclear extracts of tumour cells using kDNA as a substrate. Reaction conditions were as described above except that 1.0 μg of kDNA was used as substrate instead of pBR322 DNA. The reaction was initiated by the addition of crude

 © Cancer Research Campaign 1997

**Figure 2** Inhibitory effect of NK109 on catalytic DNA relaxation by purified yeast DNA Topo II. The enzyme activity was determined by the relaxation of supercoiled DNA (pBR322 DNA) as described in Materials and methods. Reaction mixtures (20 μl each) containing NK109 or etoposide at concentrations of 0.05–100 μg ml$^{-1}$ were incubated at 37°C for 30 min. The electrophoresis was performed on 1.0% agarose gel for 3 h nuclear extracts (0.001–1.0 μg ml$^{-1}$) and incubated at 30°C for 15 min. The subsequent process was carried out as described above.

### Cleavage of DNA by Topo II

All DNA cleavage reactions used Topo II (10 μg ml$^{-1}$) and negatively supercoiled pBR322 DNA (50 μg ml$^{-1}$) in a total volume of 20 μl of cleavage buffer (10 mM *Tris*-HCl, pH 7.9, 50 mM sodium chloride, 50 mM potassium chloride, 0.1 mM EDTA and 2.5% glycerol) that contained 5 mM magnesium chloride. Samples were incubated for 6 min at 30°C. Cleavage products were trapped (Liu et al, 1983) by the addition of 2 μl of 10% SDS. An aliquot (1 μl) of 250 mM EDTA and 2 μl of a 0.8 mg ml$^{-1}$ solution of proteinase K was added, and samples were incubated at 37°C for 30 min to digest the Topo II. Final products were mixed with 2.5 μl of loading buffer (60% sucrose, 0.05% bromophenol blue, 0.05% xylene cyanole FF, and 10 mM *Tris*-HCl, pH 7.9), heated at 70°C for 1 min, and subjected to electrophoresis in 1% agarose (MCB) gels in 40 mM Tris-acetate and 2 mM EDTA at 4 V cm$^{-1}$. After electrophoresis; DNA bands were stained with ethidium bromide

**Figure 3** NK109-induced cleavage activity of purified DNA Topo II. Reaction mixtures (20 μl each) containing purified DNA Topo II and pBR322 DNA and NK109 or etoposide at concentrations of 25 and 50 or 12.5 and 25 μg ml$^{-1}$, respectively, were incubated at 37°C for 10 min, and then were terminated by addition of 4 μl of 5% SDS and proteinase K at 2 mg ml$^{-1}$ (SDS/PK). Proteinase K digestion was performed at 37°C for 30 min. Reaction mixtures were then analysed by 1.0% agarose gel electrophoresis. The resulting bands were identified as relaxed, linear and supercoiled forms of various topological isomers of pBR322 DNA

and visualized by transillumination with ultraviolet light (300 nm). The bands were photographed through Kodak 24A and 12 filters using Polaroid type 665 positive–negative film.

### Drug accumulation study

The accumulation of [$^3$H]NK109 in the cells was determined by a previously described method (Kanzawa et al, 1990, 1995). For evaluation of drug accumulation, exponentially growing cells were harvested, 2 × 10$^7$ cells in 0.98 ml of culture medium were preincubated at 37°C in a water bath for 45 min and the cells were then exposed to 75 μM [$^3$H]NK109 at 37°C in a humidified incubator with 5% carbon dioxide. After various incubation times, the cells were collected by low-speed centrifugation, washed once with ice-cold PBS and centrifuged. The resulting cell pellet was dissolved in 0.5 ml of 90% formic acid. Clear-sol TM 1 solution (3 ml; Nacalai Tesque, Kyoto, Japan) was added to each tube and the radioactivity was measured using a liquid scintillation counter (LS6000TA, Beckman Instruments, Irvine, CA, USA). For the efflux study, cells were incubated with RPMI-FBS containing 75 μM [$^3$H]NK109 at 37°C for 60 min before being washed twice with drug-free medium. The subsequent process was then carried out as described above.

### Detection of P-glycoprotein expression by flow cytometry

For flow cytometric analysis (Nishio et al, 1990), exponentially growing AdrR MCF7 and K562/ADM cells (10$^6$) were washed with PBS and incubated for 30 min with 50 μl of anti-human P-gp monoclonal antibody (MRK-16, 10 ng ml$^{-1}$) at 4°C and used as isotype controls. The cells were washed three times with cold PBS and incubated for a further 30 min with 50 μl of fluorescein isothiocyanate (FITC) at 4°C. The fluorescence intensity of each cell line preparation was measured by flow cytometry (Hewlett Packard model 9000-340 computer interfaced with FACScan).

### Analysis of cell-surface glycoproteins

Membrane vesicles were separated by the method of Ishikawa and Ali-Osman (1993). Membrane vesicles were incubated with 0.75 μM [$^3$H]azidopine (53 Ci mmol$^{-1}$) for 15 min at room temperature in the presence or absence of various drugs. After continuous irradiation at 366 nm for 20 min at 25°C, samples were solubilized in an SDS sample buffer and run on a 7.5% SDS–polyacrylamide gel containing 4.5 M urea at a constant current of 20 mA for about

© Cancer Research Campaign 1997

© Cancer Research Campaign 1997

**Figure 4** Anti-tumour effects of NK109, doxorubicin, cisplatin and etoposide on the growth of several human cancer cell lines and their corresponding drug-resistant cells. The anti-tumour activity was measured using the regrowth assay, described in Materials and methods. Exponentially growing cells ($2 \times 10^4$ cells $ml^{-1}$) were incubated in medium containing the drug at the indicated concentration at 37°C for 7 days, after which the cells were counted and cell growth ratios were calculated. **A**, **B** and **C** show concentration–response curves of NK109 and doxorubicin against parental cells (○) and resistant cells (●) of the K562, MCF7 and SBC-3 cell lines respectively. **D** and **E** show concentration–response curves of NK109 and cisplatin against parental cells (○) and resistant cells (●) of the PC-9 and PC-14 cell lines respectively. **F** and **G** show concentration–response curves of NK109 and etoposide against parental cells (○) and resistant cells (●) of the SKOV3 and H69 cell lines respectively

4 h (Bruggemann et al, 1989). The gels were placed in Amplify (Amersham) for 30 min, dried and subjected to autoradiography at −70°C using Kodak XAR film.

### In vivo anti-tumour effect assay

Female SCID mice (5 weeks old, weighing approximately 20 g) were purchased from Nihon Clea (Tokyo, Japan) and were maintained in a room with a constant temperature (24 ± 1°C) and relative humidity (70 ± 2%), with 12 h of light a day, and were fed an X-ray irradiated commercial diet (CMF; Oriental Yeast, Tokyo, Japan) and sterilized water ad libitum, in accordance with the guidelines of our institute (Kondo et al 1994). All animals were inoculated with exponentially growing tumour cells ($3 \times 10^7$ cells per 0.05 ml) of the PC-14/P or PC-14/CDDP cell lines, which were injected subcutaneously into the right flank using an 18 G needle. When the tumour volumes increased to 500–1000 $mm^3$, 15–20 days after inoculation, the mice were divided randomly into several groups (six mice per group), in which the average tumour volumes were almost equal.

NK109 at various doses (100, 125, and 150 mg $kg^{-1}$) dissolved in physiological saline was injected intraperitoneally (i.p.) on days 0, 4 and 8. Cisplatin at 4 mg $kg^{-1}$ was injected as positive control in order to evaluate the degree of resistance to cisplatin in PC-14/CDDP cells. After drug treatment, survivors were ascertained every day during the experiment and the anti-tumour activity was

**Figure 5** Uptake of NK109 by K562/P and K562/ADM cells. Exponentially growing cells ($2 \times 10^7$ cells per test) were preincubated at 37°C for 45 min and then incubated with [$^3$H]NK109 at 37°C for the indicated time, after which the cells were collected, washed with PBS, and dissolved in formic acid. Then the radioactivity was measured with a liquid scintillation counter. ○ and □ show uptake of NK109 by K562/P and K562/ADM cells respectively. Efflux of NK109 was determined from the decline in the NK109 content of cells, accumulated during the [$^3$H]NK109 incubation, in drug-free medium for the indicated times. ● and ■ show efflux of NK109 from K562/P and K562/ADM cells respectively evaluated by the increase in lifespan (ILS), which was calculated by subtracting from 100 the percentage of the ratio of the mean survival times of treated vs untreated groups.

### RESULTS

### Catalytic activity of DNA Topo II

First, we confirmed the inhibiting effect of NK109 on DNA Topo II catalytic activity that was reported to be the mechanism of cytotoxicity of NK109 (Kabasawa et al, 1996). Catalytic activity of Topo II was assayed by relaxation of supercoiled pBR322 DNA and the result is shown in Figure 2. In the absence of Topo II, pBR322 DNA migrates predominantly in the fast moving supercoiled state. Addition of Topo II without drug results in conversion of the supercoiled form into the relaxed DNA configurations. NK109 inhibited the ability of Topo II to relax supercoiled DNA at 5 μg ml$^{-1}$ However, at concentrations of less than 20 μg ml$^{-1}$, NK109 did not inhibit the relaxation. As a reference compound, etoposide also inhibited the relaxation of pBR322 DNA by Topo II at the same concentration as shown in Figure 2. NK109 inhibits the catalytic activities of purified DNA Topo II in vitro, and its efficacy is as high as etoposide.

### Effect of NK109 on the DNA cleavage of topo II

The effect of NK109 on the DNA cleavage of Topo II was studied by 25–50 μg ml$^{-1}$ drug in reaction mixtures (Figure 3). Topo II-mediated DNA strand passage requires breaking and rejoining of the double-stranded DNA. During this process, the enzyme becomes covalently linked to the 5′-phosphate of both DNA strands via a tyrosine–DNA phosphodiester of both DNA strands via a tyrosine–DNA phosphodiester linkage (Tse et al, 1980). The covalent reaction intermediate is called the cleavable complex and can be demonstrated experimentally by the enzyme-dependent formation of linear DNA from supercoiled DNA after treatment with SDS and proteinase K (Liu et al, 1983). Clinically used DNA topoisomerase II inhibitors such as doxorubicin and etoposide act by stabilizing the cleavable complex (Nelson et al, 1984; Ross et al, 1984; Tewey et al, 1984). NK109 at concentrations up to 10 μg ml$^{-1}$ did not induce DNA cleavage (data not shown). In contrast, 25 μg ml$^{-1}$ NK109 completely inhibited the cleavable complex formation as shown in Figure 3. A similar effect was also observed in the treatment using etoposide. These results suggest that DNA Topo II is a target of NK109 action and this action may play a role in the cytotoxicity of NK109.

### In vitro growth-inhibiting activity of NK109 in drug-resistant cells

We examined whether NK109 showed cytotoxicity against a panel of multidrug-resistant cell lines, such as K562/ADM, AdrR MCF7, SBC-3/ADM and H69/VP. The sensitivity or resistance was verified by regrowth assays in a continuous drug exposure system. Resistance indices were determined by comparing the toxic doses allowing 50% survival ($IC_{50}$) of resistant cell lines with their parent cell lines. Resistance indices of K562/ADM, AdrR MCF7 and SBC-3/ADM were 59.8-, 62.0- and 10.2-fold as shown in Figure 4A–C respectively. The cytotoxicity of NK109 against these three doxorubicin-resistant cell lines was determined. The $IC_{50}$ of NK109 for K562/ADM cells was 0.045 μg ml$^{-1}$, which is almost the same as that (0.041 μg ml$^{-1}$) of the parent cell line, suggesting no cross-resistance to NK109, as shown in Figure 4A. The $IC_{50}$ of NK109 for AdrR MCF7 cells was 0.42 μg ml$^{-1}$ which is 2.1 times that (0.20 μg ml$^{-1}$) of the parent cell line (Figure 4B). SBC-3/ADM cells were almost as sensitive to NK109 as SBC-3/P cells (Figure 4C). The etoposide-resistant H69/VP cell line, having a typical P-gp-mediated MDR phenotype, also showed no cross-resistance to NK109 (Figure 4G).

Cisplatin-resistant PC-9/CDDP and PC-14/CDDP human non-small-cell lung cancer cells were examined for cross-resistance to NK109. The resistance ratios of PC-9/CDDP cells and PC-14/CDDP cells for cisplatin were 4.4- and 12.9-fold respectively. The cytotoxicities of NK109 against these cisplatin-resistant cell lines were estimated. The $IC_{50}$ of NK109 for PC-9/CDDP cells was 0.19 μg ml$^{-1}$, which is almost the same as for the parental cell line (0.17 μg ml$^{-1}$) (Figure 4D). PC-14/CDDP cells showed no cross-resistance to NK109 as shown in Figure 4E. Thus, NK109 was effective against cisplatin-resistant PC-9 and PC-14 cells as well as against their parental cells.

We postulated that NK109 would not inhibit the growth of etoposide-resistant cells because NK109 is thought to exert its cytotoxicity by the formation of cleavable Topo II complexes, as does etoposide. SKOV3/VP cells are 9.6-fold resistant to etoposide and decreased Topo II activity is the resistance mechanism (Kubota et al, 1994). the $IC_{50}$ of NK109 for the growth of SKOV3/VP cells was 0.21 μg ml$^{-1}$, which is little higher than that of the parental cells (Figure 4F). Thus, contrary to expectation, we did not observe cross-resistance of SKOV3/VP cells to NK109.

### Intracellular accumulation of NK109 in drug-resistant cells

We studied reactions of NK109 with several molecular and biological alterations associated with resistance. First, the time course of NK109 uptake by K562 cells was determined, because resistance to anti-tumour drugs is frequently characterized by diminished

© Cancer Research Campaign 1997

**Figure 6** P-glycoprotein expression in K562/ADM and AdrR MCF7 cell lines. In each overlay histogram, the horizontal axis records fluorescein isothiocyanate fluorescence of MRK-16-treated or isotype control cells and the vertical axis represents the number of cells. Histograms in black and those in white are of MRK-16-treated cells and the isotype controls respectively. Numbers of P-glycoprotein-positive cells, after subtraction of fluorescent cells from the isotype control, are recorded as a percentage. (**A**) K562/ADM; (**B**) AdrR MCF7

drug accumulation in resistant cells. When the cells were incubated with 75 μM [$^3$H]NK109 (0.5 μCi), the cellular uptake of NK109 into K562/ADM cells was the same as that in the parental cells. Further, we determined the efflux of NK109 from the resistant cells compared with the parent cells and the results are shown (● and ■ for K562/P and K562/ADM respectively) in Figure 5. Efflux did not differ between the cell lines, as shown in Figure 5.

The mechanism of resistance of the cisplatin-resistant PC-14/CDDP cell line is thought to be impaired drug uptake, so we determined the cellular uptake of NK109 by PC14/P and PC14/CDDP cells. No difference in the uptake of NK109 between these cell lines was observed (data not shown).

### Detection of P-gp in doxorubicin-resistant cells

The K562/ADM and AdrR MCF7 cells were confirmed to be P-gp positive. Flow cytometric overlay histograms of isotype controls and MRK-16 antibody-treated cells are shown in Figure 6. In the parental cell lines K562 and MCF7, less than 1% of cells stained positive with the P-gp-specific MRK-16 monoclonal antibody (data not shown). In contrast, both K562/ADM and AdrR MCF7 cells reacted strongly with MRK-16 (Figure 6A and 6B). SBC-3/ADM and H69/VP cells also exhibited P-gp (data not shown).

### Binding activity of NK109 with P-gp

P-gp is a plasma membrane protein that confers MDR by actively transporting the cytotoxic drugs out of the cells. Therefore, we examined the affinity of NK109 for P-gp. Azidopine is a photoaffinity analogue of the dihydropyridine class of calcium channel blockers that specifically labels P-gp (Yang et al, 1988). Verapamil, a calcium channel blocker, inhibited this labelling, as shown in Figure 7. Doxorubicin also inhibited photolabelling of P-gp by [$^3$H] azidopine. This result suggests that both verapamil and doxorubicin are substrates for active transport in plasma membrane vesicles prepared from multidrug-resistant K562 cells. In contrast, NK109 does not block photolabelling when present at up to a 100-fold molar excess (Figure 7), suggesting that NK109 is not a substrate for P-gp and, therefore, that accumulation of NK109 was not diminished in resistant cells.

© Cancer Research Campaign 1997 

**Figure 7** Inhibition of [$^3$H]azidopine labelling of P-glycoprotein in membrane vesicles from K562 cells by NK109 and the reference agents verapamil and doxorubicin. K562 cell vesicles were incubated with [$^3$H]azidopine in the absence or presence of the indicated concentration of the analogues. P-glycoprotein was analysed by sodium dodecyl sulphate–urea–polyacrylamide electrophoresis. Photographs show autoradiograms developed after 30 days' exposure. Graphs show results quantified by scanning densitometry

### Topo I and Topo II activities of etoposide-resistant cells

The total cellular Topo II activities of crude nuclear extracts eluted with 0.35 M sodium chloride from SKOV3/P cells and their etoposide-resistant sublines were measured. The decatenation of kDNA incubated with different amounts of SKOV3/P and SKOV3/VP nuclear protein extracts is shown in Figure 8B. Decatenated forms were not observed for SKOV3/VP even when 0.03 μg of nuclear extract was used, indicating that Topo II activity of SKOV3/VP cells was reduced to about one-tenth of that of the parent cells. In contrast, the Topo I activity of SKOV3/VP extracts was not changed compared with the parent cells (Figure 8A). These results indicate that the resistance of SKOV3/VP cells to etoposide is due to reduced Topo II activity. Non-cross-resistance of etoposide-resistant SKOV3/VP cells to NK109 suggests that the mechanism of action of NK109 differs from that of etoposide.

### In vivo anti-tumour activity of NK109 against drug-resistant cells

In order to confirm the in vitro results of non-cross-resistance to NK109 in drug-resistant cells, we checked the transplantability of several human carcinoma cell lines in nude or SCID mice. We found that both PC-14/P and PC-14/CDDP were transplantable in SCID mice, producing high take rates, and thus SCID mice were used in this experiment.

Figure 9B shows the mean ILS of cisplatin-treated SCID mice with PC-14/P and PC-14/CDDP cell xenografts. The cisplatin treatment suppressed tumour growth only in the PC-14/P cell line and not in the resistant subline PC-14/CDDP. Therefore, the ILSs for cisplatin (4 mg $kg^{-1}$) for PC-14/P and PC-14/CDDP cells were 44% and 2% respectively. In contrast, the ILSs for NK109 at 100, 125, and 150 mg $kg^{-1}$ for PC-14/P cells were 8%, 26% and 33% respectively. The ILSs for PC-14/CDDP cells were 18%, 25% and 27% respectively as shown in Figure 9A. These data indicate that cisplatin-resistant cells are not cross-resistant to NK109 in vivo, as well as in vitro.

### DISCUSSION

Fagaridine isolated from the root bark of *Fagara xanthoxyloides* was reported to inhibit the growth of certain human leukaemias and several murine tumour cells, and 2,3-(methylenedioxy)-5-methyl-7-hydroxy-8-methoxybenzo[c]phenanthridinium was determined as its chemical structure, which is similar to that of NK109 (Torto and Mensah, 1973; Hanaoka et al, 1985). However, Kessar et al (1988) indicated an incorrect structure for fagaridine and revised a different placement (at the C-8 position on ring A of benzo[c]phenanthridine) of the hydroxy group. Therefore, the NK109 tested in this study is a different positional isomer from fagaridine and a novel anti-tumour agent.

Its cytotoxic mechanism was confirmed to be due to Topo II inhibition. The topoisomerases are nuclear enzymes that catalyse the concerted breaking and rejoining of DNA strands, thereby controlling the topological states of DNA during many DNA metabolic processes, including replication, recombination, transcription and chromosome segregation at mitosis (Wang, 1985). They are considered to be important therapeutic targets in cancer chemotherapy. In addition, recent studies have shown that several chemotherapeutic drugs, such as topoisomerase inhibitors, trigger apoptosis (Kaufmann, 1989). We have also demonstrated that NK109 induces single- and double-strand DNA breaks and DNA fragmentation, which is a marker for apoptosis, in a human small-cell lung cancer cell line. Furthermore, we showed that protein and RNA syntheses were not required for apoptosis induced by NK109 (Fukuda et al, 1996).

In this study, we determined the efficacy of NK109 against an etoposide-resistant ovarian carcinoma cell line, SKOV3/VP, which has reduced Topo II activity as one of the resistance mechanisms. The resistant cells exhibited marked sensitivity to NK109 compared with their parent cells, suggesting that the cytotoxic mechanism of NK109 differs from that of etoposide. This was supported by the observation that NK109 induces more DNA strand breaks, especially DNA double-strand breaks, than the same concentration of etoposide (Fukuda et al, 1996). Wang et al

© Cancer Research Campaign 1997

**Figure 8** Topo I (**A**) and Topo II (**B**) activities of SKOV3/P and SKOV3/VP cells. The enzymic activity of Topo I was determined by measuring the relaxation of supercoiled pBR 322 DNA after incubation with nuclear extract (0.0003–1.0 µg) at 37°C for 20 min, using electrophoresis on a 0.7% agarose gel for 4.5 h. The Topo II catalytic activity was determined by decatenation of kDNA. The reaction mixture, containing 1 µg of kDNA and nuclear extract (0.001–1.0 µg), was incubated at 30°C for 15 min. The subsequent process was carried out as described above in the electrophoresis

**Figure 9** Increase in lifespan (ILS) of SCID mice with PC-14/P (○) and PC-14/CDDP (●) cell xenografts, treated by injection (i.p.) of NK109 at doses of 100, 125 and 150 mg kg$^{-1}$ (**A**) and cisplatin at a dose of 4 mg kg$^{-1}$ (**B**) as a reference drug on days 0, 4 and 8. ILS was calculated as described in Materials and methods

(1993) reported that fagaronine and nitidine being similar to NK109 are characterized as inhibitors of Topo I function. Therefore, we are studying the ability of NK109 to induce single-strand DNA breaks or act as a Topo I poison.

The development of multiple resistance to anti-tumour agents by human tumour cells is recognized as one of the major obstacles to successful cancer chemotherapy. MDR is frequently characterized by enhanced drug efflux because of a membrane glycoprotein (P-gp; $M_r$ 170 000) encoded by the *MDR1* gene in human cancer cells (Fojo et al, 1985). The kinetics of drug uptake, accumulation and efflux also suggests that multidrug-resistant cells remove these cytotoxic drugs from the cell by active transport across the plasma

© Cancer Research Campaign 1997

membrane (Skovsgaard, 1978). Overexpression of P-gp is widely observed in various multidrug-resistant cell lines (Dano, 1973). Further, multidrug-resistant cells selected for resistance to one drug become simultaneously resistant to many other drugs that are structurally and functionally unrelated to the selected drug (Gottesman and Pastan, 1988). For instance, human KB carcinoma cells selected for resistance to colchicine are also resistant to vinblastine, vincristine, actinomycin D, daunomycin and doxorubicin, but they are not resistant to methotrexate, cytosine arabinoside and dexamethasone (Fojo et al, 1985).

Some calcium channel blockers, such as verapamil, diltiazem, and dihydropyridine analogues, are reported to reverse the MDR phenotype (Safa et al, 1987; Akiyama et al, 1988). However, no agent that reverses multidrug resistance can be clinically used in full doses to reverse resistance. Thus, the development of NK109 as a novel anti-tumour drug that is not a substrate for calcium channel blockers of P-gp will be potentially valuable. Moreover, the efficacy of NK109 is not susceptible to several molecular alterations associated with drug resistance. The clinical study of this compound is now in progress in Japan. The drug is clearly very active against resistant solid tumour cell lines, but its clinical toxicity needs elucidation before human clinical efficacy trials.

### ACKNOWLEDGEMENTS

This work was supported, in part, by Grants-in-Aid for Cancer Research and from the Second Term Comprehensive 10-Year Strategy for Cancer Control, the Ministry of Health and Welfare, the Ministry of Education and Science of Japan, a trust fund, the Adult Disease Memorial foundation and Nippon Kayaku (Tokyo, Japan). The authors gratefully acknowledge the kind advice of Dr Andrew Turrisi, Professor and Chairman. Department of Rad Oncol. Med., University South Carolina. This work was supported by Grants-in-Aid for cancer research from the Comprehensive Ten-Year Strategy for Cancer Control, from the Ministry of Health and Welfare, Japan.

### ABBREVIATIONS

Topo I, DNA topoisomerase I; topo II, DNA topoisomerase II; kDNA, kinetoplast DNA; PBS, phosphate-buffered saline; PMSF, phenylmethylsulphonyl fluoride; SDS, sodium dodecyl sulphate; NB, nuclear buffer.

### REFERENCES

Akiyama S, Cornwell MM, Kuwano M, Pastan I and Gottesman MM (1988) Most drugs that reverse multidrug resistance also inhibit photoaffinity labeling of P-glycoprotein by a vinblastine analog. *Mol Pharmacol* **33**: 144–147

Barret Y and Sauvaire Y (1992) Fagaronine, a novel antileukemic alkaloid. *Phytother Res* **6**: 59–63

Bruggemann EP, Germann UA, Gottesman MM and Pastan I (1989) Two different regions of P-glycoprotein [corrected] are photoaffinity-labeled by azidopine [published erratum appears in *J Biol Chem* 1990. **265**: 4172]. *J Biol Chem* **264**: 15483–15488

Cowan KH, Batist G, Tulpule A, Sinha BK and Myers CE (1986) Similar biochemical changes associated with multidrug resistance in human breast cancer cells and carcinogen-induced resistance to xenobiotics in rats. *Proc Natl Acad Sci USA* **83**: 9328–9332

Dano K (1973) Active outward transport of daunomycin in resistant Ehrlich ascites tumor cells. *Biochim Biophys Res Acta* **323**: 466–483

Deffie AM, Batra JK and Goldenberg GJ (1989) Direct correlation between DNA topoisomerase II activity and cytotoxicity in adriamycin-sensitive and -resistant P388 leukemia cell lines. *Cancer Res* **49**: 58–62

Fojo A, Akiyama S, Gottesman MM and Pastan I (1985) Reduced drug accumulation in multiply drug-resistant human KB carcinoma cell lines. *Cancer Res* **45**: 3002–3007

Fukuda M, Inomata M, Nishio K, Fukuoka K, Kanzawa F, Arioka H, Ishida T, Fukumoto H, Kurokawa H, Oka M and Saijo N (1996) A topoisomerase II inhibitor, NK109, induces DNA single- and double-strand breaks and apoptosis. *Jap J Cancer Res* **87**: 1086–1091

Gottesman MM and Pastan I (1988) The multidrug transporter, a double-edged sword (Review). *J Biol Chem* **263**: 12163–12166

Hanaoka M, Yamagishi H and Murai C (1985) Synthesis of fagaridine, a phenolic benzo[c]phenanthridine alkaloid. *Chem Pharm Bull* **33**: 1763–1765

Hong WS, Saijo N, Sasaki Y, Minato K, Nakano H, Nakagawa K, Fujiwara Y, Nomura K and Twentyman PR (1988) Establishment and characterization of cisplatin-resistant sublines of human lung cancer cell lines. *Int J Cancer* **41**: 462–467

Horichi N, Tapiero H, Sugimoto Y, Bungo M, Nishiyama M, Fourcade A, Lampidis TJ, Kasahara K, Sasaki Y, Takahashi T and Saijo N (1990) 3′-Deamino-3′-morpholino-13-deoxo-10-hydroxycarminomycin conquers multidrug resistance by rapid influx following higher frequency of formation of DNA single- and double-strand breaks. *Cancer Res* **50**: 4698–4701

Ishikawa T and Ali-Osman F (1993) Glutathione-associated cis-diamminedichloroplatinum(II) metabolism and ATP-dependent efflux from leukemia cells. Molecular characterization of glutathione-platinum complex and its biological significance. *J Biol Chem* **268**: 20116–20125

Kabasawa T, Kobayashi F, Ekimoto H, Suzuki M and Hanaoka M (1996) A novel benzo[c]phenanthridine derivative, NK109, is a highly active anticancer agent and DNA topoisomerase II inhibitor. *Proc Am Assoc Cancer Res* **37**: 427

Kanzawa F, Hoshi A, Kuretani K, Saneyoshi M and Kawaguchi T (1981) Antitumor activity of 3′,5′-diesters of 5-fluoro-2′-deoxyuridine against murine leukemia L1210 cells. *Cancer Chemother Pharmacol* **6**: 19–23

Kanzawa F, Maeda M, Sasaki T, Hoshi A and Kuretani K (1982) Murine lymphoma L5178Y cells resistant to purine antagonists: differences in cross-resistance to thioguanine-platinum(II) and selenoguanine-platinum(II). *J Natl Cancer Inst* **68**: 287–291

Kanzawa F, Sugimoto Y, Minato K, Kasahara K, Bungo M, Nakagawa K, Fujiwara Y, Liu LF and Saijo N (1990) Establishment of a camptothecin analogue (CPT-11)-resistant cell line of human non-small cell lung cancer: characterization and mechanism of resistance. *Cancer Res* **50**: 5919–5924

Kanzawa F, Nishio K, Kubota N and Saijo N (1995) Antitumor activities of a new indolocarbazole substance, NB-506, and establishment of NB-506-resistant cell lines, SBC-3/NB. *Cancer Res* **55**: 2806–2813

Kaufmann SH (1989) Induction of endonucleolytic DNA cleavage in human acute myelogenous leukemia cells by etoposide, camptothecin, and other cytotoxic anticancer drugs: a cautionary note. *Cancer Res* **49**: 5870–5878

Kessar SV, Gupta YP, Balakrishnan P, Sawal KK, Mohammad T and Dutt M (1988) Benzyne cyclization route to benzo[c]phenanthridine alkaloids. Synthesis of chelerythrine, decarine, and nitidine. *J Org Chem* **53**: 1708–1713

Kimura I, Ohnoshi T, Hiraki S and Miyamoto H (1987) Establishment of adriamycin-resistant human small-cell lung cancer cells in culture: analysis of the mechanism of resistance and cross-resistance. *Jpn J Cancer Chemother* **14**: 830–836

Kondo H, Kanzawa F, Nishio K, Saito S and Saijo N (1994) In vitro and in vivo effects of cisplatin and etoposide in combination on small cell lung cancer cell lines. *Jpn J Cancer Res* **85**: 1050–1056

Kubota N, Nishio K, Takeda Y, Ohmori T, Funayama Y, Ogasawara H, Ohira T, Kunikane H, Terashima Y and Saijo N (1994) Characterization of an etoposide-resistant human ovarian cancer cell line. *Cancer Chemother Pharmacol* **34**: 183–190

Larsen AK, Grondard L, Couprie J, Desoize B, Comoe L, Jardillier JC and Riou JF (1993) The antileukemic alkaloid fagaronine is an inhibitor of DNA topoisomerases I and II. *Biochem Pharmacol* **46**: 1403–1412

Liu LF and Miller KG (1981) Eukaryotic DNA topoisomerases: two forms of type I DNA topoisomerases from HeLa cell nuclei. *Proc Natl Acad Sci USA* **78**: 3487–3491

Liu LF, Rowe TC, Yang L, Tewey KM and Chen GL (1983) Cleavage of DNA by mammalian DNA topoisomerase II. *J Biol Chem* **258**: 15365–15370

Marini JC, Miller KG and Englund PT (1980) Decatenation of kinetoplast DNA by topoisomerases. *J Biol Chem* **255**: 4976–4979

Minato K, Kanzawa F, Nishio K, Nakagawa K, Fujiwara Y and Saijo N (1990) Characterization of an etoposide-resistant human small-cell lung cancer cell line. *Cancer Chemother Pharmacol* **26**: 313–317

Nelson EM, Tewey KM and Liu LF (1984) Mechanism of antitumor drug action: Poisoning of mammalian DNA topoisomerase II on DNA by 4′-(9-acridinylamino)-methanesulfon-m-aniside. *Proc Natl Acad Sci USA* **81**: 1361–1365

© Cancer Research Campaign 1997

Nishio K, Sugimoto Y, Nakagawa K, Niimi S, Fujiwara Y, Bungo M, Kasahara K, Fujiki H and Saijo N (1990) Cross-resistance to tumour promoters in human cancer cell lines resistant to adriamycin or cisplatin. *Br J Cancer* **62**: 415–419

Ohmori T, Morikage T, Sugimoto Y, Fujiwara Y, Kasahara K, Nishio K, Ohta S, Sasaki Y, Takahashi T and Saijo N (1993) The mechanism of the difference in cellular uptake of platinum derivatives in non-small cell lung cancer cell line (PC-14) and its cisplatin-resistant subline (PC-14/CDDP). *Jpn J Cancer Res* **84**: 83–92

Ross W, Rowe T, Glisson B, Yalowich J and Liu LF (1984) Role of topoisomerase II in mediating epipodophyllotoxin-induced DNA cleavage. *Cancer Res* **44**: 5857–5860

Safa AR, Glover CJ, Sewell JL, Meyers MB, Biedler JL and Felsted RL (1987) Identification of the multidrug resistance-related membrane glycoprotein as an acceptor for calcium channel blockers. *J Biol Chem* **262**: 7884–7888

Skovsgaard T (1978) Mechanisms of resistance to daunorubicin in Ehrlich ascites tumor cells. *Cancer Res* **38**: 1785–1791

Tewey KM, Chen GL, Nelson EM and Liu LF (1984) Intercalative antitumor drugs interfere with the breakage–reunion reaction of mammalian DNA topoisomerase II. *J Biol Chem* **259**: 9182–9187

Torto FG and Mensah IA (1973) Fagaridine: a phenolic benzophenanthridine alkaloid from *Fagara xanthoxyloides. Phytochemistry* **12**: 2315–2317

Tse YC, Kirkegaard K and Wang JC (1980) Covalent bounds between protein and DNA. *J Biol Chem* **255**: 5560–5565

Tsuruo T, Iida, HS, Kawabata H, Oh-Hata T, Hamada H and Utakoji T (1986) Characteristics of resistance to adriamycin in human myelogenous leukemia K562 resistant to adriamycin and in isolated clones. *Jpn J Cancer Res* **77**: 682–692

Wang JC (1985) DNA topoisomerases. *Annu Rev Biochem* **54**: 665–697

Wang LK, Johnson RK and Hecht CM (1993) Inhibition of topoisomerase I function by nitidine and fagaronine. *Chem Res Toxocol* **6**: 813–818

Yang CP, Mellado W and Horwitz SB (1988) Azidopine photoaffinity labeling of multidrug resistance-associated glycoproteins. *Biochem Pharmacol* **37**: 1417–1421

Yoshida M, Feng W, Saijo N and Ikekawa T (1996) Antitumor activity of daphnane-type diterpene gnidimacrin isolated from stellera cheamaejasme L. *Int J Cancer* **66**: 268–273

© Cancer Research Campaign 1997